# Glomerular Filtration and Podocyte Tensional Homeostasis: Importance of the Minor Type IV Collagen Network

**DOI:** 10.1007/s10237-020-01347-y

**Published:** 2020-05-27

**Authors:** Lauren M. Bersie-Larson, Lazarina Gyoneva, Daniel J. Goodman, Kevin D. Dorfman, Yoav Segal, Victor H. Barocas

**Affiliations:** 1 Department of Biomedical Engineering, University of Minnesota; 2 Department of Chemical Engineering and Materials Science, University of Minnesota; 3 Division of Renal Diseases and Hypertension, Department of Medicine, University of Minnesota; 4 Minneapolis VA Health Care System

**Keywords:** Kidney, Capillary, Biomechanics, Growth, Remodeling, Stability

## Abstract

The minor type IV collagen chain, which is a significant component of the glomerular basement membrane in healthy individuals, is known to assemble into large structures (supercoils) that may contribute to the mechanical stability of the collagen network and the glomerular basement membrane as a whole. The absence of the minor chain, as in Alport syndrome, leads to glomerular capillary demise and eventually to kidney failure. An important consideration in this problem is that the glomerular capillary wall must be strong enough to withstand the filtration pressure *and* porous enough to permit filtration at reasonable pressures. In this work, we propose a coupled feedback loop driven by filtration demand and tensional homeostasis of the podocytes forming the outer portion the glomerular capillary wall. Briefly, the deposition of new collagen increases the stiffness of basement membrane, helping to stress shield the podocytes, but the new collagen also decreases the permeability of the basement membrane, requiring an increase in capillary transmural pressure drop to maintain filtration; the resulting increased pressure outweighs the increased glomerular basement membrane stiffness and puts a net greater stress demand on the podocytes. This idea is explored by developing a multiscale simulation of the capillary wall, in which a macroscopic (μm-scale) continuum model is connected to a set of microscopic (nm-scale) fiber network models representing the collagen network and the podocyte cytoskeleton. The model considers two cases: healthy remodeling, in which the presence of the minor chain allows the collagen volume fraction to be increased by thickening fibers, and Alport syndrome remodeling, in which the absence of the minor chain allows collagen volume fraction to be increased only by adding new fibers to the network. The permeability of the network is calculated based on previous models of flow through a fiber network, and it is updated for different fiber radii and volume fractions. The analysis shows that the minor chain allows a homeostatic balance to be achieved in terms of both filtration and cell tension. Absent the minor chain, there is a fundamental change in the relation between the two effects, and the system becomes unstable. This result suggests that mechanobiological or mechanoregulatory therapies may be possible for Alport syndrome and other minor-chain collagen diseases of the kidney.

## Introduction

Human glomerular capillaries, through which some 180 liters of blood are filtered daily in aggregate, face a difficult structural challenge, as discussed two decades ago by Kriz (Kriz et al. 1996): The capillary wall must be strong enough to resist the mechanical pressure driving plasma filtration, but also permeable enough to allow filtration at physiological pressures. These competing drives make the remodeling environment of glomerular capillaries fundamentally different from that in arteries and other blood vessels (Martinez-Lemus et al. 2009; Humphrey 2009; Califano and Reinhart-King 2010; Dahl et al. 2010; Ambrosi et al. 2011).

Conceptually, one can identify two coupled control processes within glomerular capillaries. The more rapid of the two, occurring in seconds, is renal autoregulation, which encompasses myogenic and tubuloglomerular feedback responses controlling renal blood flow and glomerular filtration rate (GFR) (Singh and Thomson 2010; Carlstrom et al. 2015). The glomerular filtration pressure adjusts very quickly to ensure that GFR meets physiological needs. We refer to the maintenance of a steady filtration rate by adjusting the glomerular capillary pressure as *filtration homeostasis*. The second, slow control process is glomerular capillary remodeling, which entails changes in both the cells and the extracellular matrix and has received little attention to date from the theoretical community. In this work, we focus on matrix turnover within the glomerular basement membrane (GBM), specifically that of its major constituent type IV collagen. Over months to years (Walker 1973; Cohen et al. 1982), new material is deposited by podocytes (Abrahamson et al. 2009), specialized epithelial cells that surround the GBM, and old material is degraded. If one postulates a model in which the podocytes are sensing GBM stretch, which is consistent with in vitro studies of podocyte mechanobiology, (Endlich et al. 2001; Petermann et al. 2002, 2005; Durvasula et al. 2004), and depositing collagen IV so as to reach a specific target cell stress (i.e., *tensional homeostasis*, cf. (Taber 2008; Dufort et al. 2011; Valentin et al. 2011; Webster et al. 2014)), then one must conclude that there is a risk of a positive feedback loop arising, in which newly deposited matrix decreases the permeability of the GBM by a larger factor than it increases the stiffness (Barocas et al. 2012).

The challenge of balancing stiffness and permeability has been met biologically by the so-called minor type IV collagen chain. The vast majority of type IV collagen is formed from a triple helix of the major chain (α_1_α_1_α_2_), but in basement membranes with important mechanical functions, such as the GBM (Gunwar et al. 1998; Hudson et al. 2003) or the ocular lens capsule (Kelley et al. 2002), a minor chain (α_3_α_4_α_5_) is present in significant amounts. By virtue of its more frequent Gly-X-Y interruptions, some containing cysteine, the minor chain is both more flexible and more able to form disulfide bonds, leading to stronger interactions between chains and supercoiling of different triple helices. Inability to synthesize the minor chain as a result of genetic mutations leads to Alport syndrome, which is characterized by kidney failure through demise of glomerular capillaries (Jais et al. 2000; Hudson et al. 2003; Rheault et al. 2004). Thin basement membrane disease is a related disorder that is marked by partial synthesis of the minor chain and generally less severe. These and other disorders of the type IV collagen minor chain are all alternatively classified as Alport syndrome encompassing an expanded spectrum of clinical and genetic features (Kashtan et al. 2018).

We showed previously that supercoiling can lead to increased permeability without sacrificing stiffness (or, equivalently, increased stiffness without sacrificing permeability), a concept that would be consistent with the minor chain’s presence in the GBM and its possible role in allowing the GBM to maintain both strength (or stiffness) and permeability (Gyoneva et al. 2015). Such a role would be evinced by differences between remodeling behavior in the presence of both the major and the minor chain (i.e., in a computational model representing healthy GBM) and remodeling behavior absent the minor chain (representing Alport syndrome). In the current work, we further develop that idea and consider whether the ability of the minor chain to supercoil allows the GBM to achieve both tensional and filtration homeostasis.

## Methods

### Structural Multiscale Model of the Glomerular Capillary Wall

**Figure 1.**
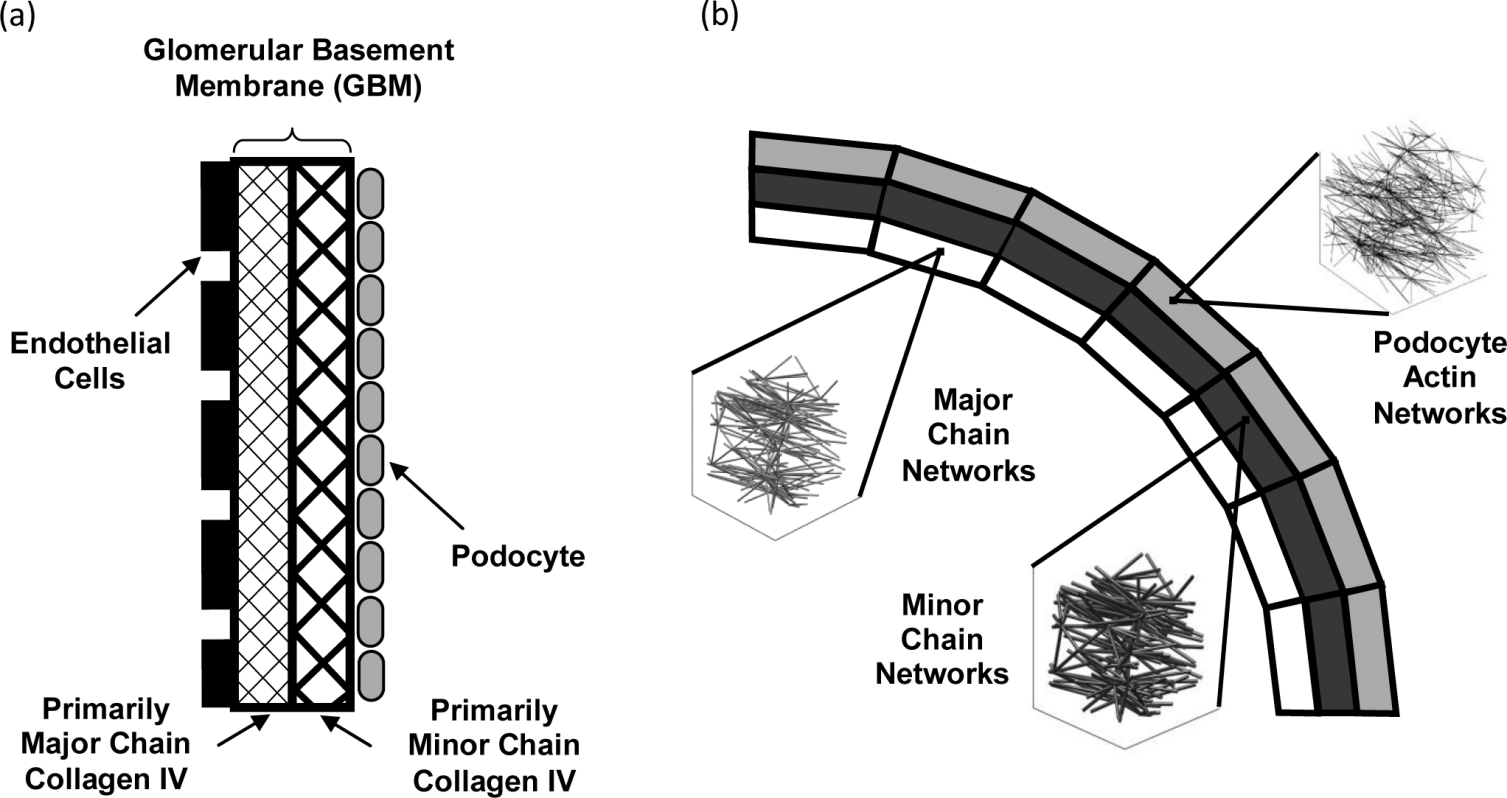
Model Schematic. (a) Conceptual schematic of the glomerular capillary wall, consisting of four distinct layers from the innermost endothelial layer to the outermost podocyte (epithelial) layer. (b) Finite element model geometry consisting of a quarter cylinder containing major-chain, minor-chain, and podocyte layers, each represented using our multiscale modeling scheme and underlying collagen IV networks (for GBM) or actin filament networks (for podocytes).

Figure 1a shows a schematic of the glomerular capillary wall, consisting of four layers:

- A fenestrated endothelium on the inner (luminal) surface,
- An inner portion of the GBM that is predominantly major-chain collagen,
- An outer portion of the GBM having significant minor-chain collagen content, and
- A podocyte layer on the outer (urinary) surface.

Although the glomerular capillary’s fenestrated endothelium plays a role in glomerular filtration (see (Haraldsson et al. 2008) for discussion), it is not known to remodel and thus is not included in the current model. The model consists of a quarter (by symmetry) of a ring-shaped slice of the glomerular capillary wall (Figure 1b). The distinct inner (major-chain) and outer (minor-chain) GBM layers are based on the known generation of first major chain and then minor chain collagen during development (Miner and Sanes 1994) and the fact that new matrix deposition occurs primarily in the outer portion of the GBM (Walker 1973); in Alport syndrome, the minor chain cannot be produced, so both layers are treated as major chain only. The model treats the segregation of the major and minor chains into different layers as perfect (i.e., one layer of pure major chain and one of pure minor chain). Around the outer surface of the vessel, a third layer is introduced, representing the podocytes; this simplification of the podocyte’s contribution to GBM mechanics and the filtration process (Drumond and Deen 1994; Edwards et al. 1997; Haraldsson et al. 2008), permits inclusion of the podocytes and an estimate of the stress experienced by the podocytes as a result of transcapillary pressure.

For solid mechanics, the modeling scheme is similar to that which we have used previously in other settings (Stylianopoulos and Barocas 2007; Lai et al. 2013; Zarei et al. 2017). The macroscopic (tissue) scale is represented by a standard finite-element mesh, but instead of a closed-form constitutive equation, a representative volume element (RVE) containing a unique, randomly-generated fiber network is introduced at each Gauss point to represent the mechanics at the microscopic (collagen or cytoskeletal) scale. Displacements from the macroscopic scale are passed down to each RVE and imposed affinely on the RVE boundaries, and the fiber network within the RVE is then allowed to rearrange (non-affinely) so as to balance force at each network node. The volume-averaged stress in each RVE is calculated and passed back up to the tissue scale, and the process iterates until both the macroscopic and microscopic scales are both at mechanical equilibrium. Further details on this approach are available in our earlier papers (Chandran and Barocas 2007; Chandran et al. 2008). All fiber networks were created by placing random seed points and then connecting them in a Delaunay triangulation using the Matlab *delaunay* function. Fiber mechanics were governed by the following equation for fiber force *f*:

(1) $$f=\frac{AE}{B}[\text{exp}(B\varepsilon )-1]$$

where A is the cross-sectional area of the fiber, E is the fiber modulus, B is a fitting parameter related to fiber nonlinearity, and ε is defined as the Green strain, expressed in terms of the fiber stretch λ as

(2) $$\varepsilon =\frac{1}{2}\left(\lambda^{2}-1\right)$$

The parameter values used in the model are given in Table 1. The boundary conditions on the model are symmetry on the horizontal and vertical cuts, urinary pressure on the outer surface, and the glomerular filtration pressure in the inner surface of the cylindrical capillary wall.

The permeability of the GBM layers was calculated from the fiber networks following (Stylianopoulos et al. 2008). First, for each fiber in an RVE a local drag coefficient tensor was calculated based on established expressions (Sangani and Acrivos 1982; Drummond and Tahir 1984) for per-cylinder drag on a regular array of cylinders, where the volume fraction is set based on the known fiber volume fraction in the RVE. That is, for each fiber, a contribution to the overall drag on fluid flowing through the network was calculated as if the network was replaced with a regular array of fibers all pointing in the same direction as the fiber in question. The resulting drag coefficient is a tensor, not a scalar, because flow parallel to a fiber generates considerably less drag than flow perpendicular to a fiber.

Next, the local tensor for each fiber was expressed in the RVE coordinate system, and all of the local drag coefficient tensors were summed to give an overall drag coefficient tensor for the RVE. This drag coefficient tensor was then inverted to give an RVE permeability tensor, and the total GBM permeability was obtained as the harmonic mean of the radial components of the inner and outer layer permeability tensors, consistent with permeabilities in series.

### Remodeling of the GBM – Major vs. Minor Chain Remodeling

For the purpose of this analysis, it is assumed that there is no major organ-level change to the kidney (i.e., the number of functional nephrons does not change), so that the total glomerular filtration rate, the single-nephron glomerular filtration rate, and the glomerular filtration velocity are all assumed to be maintained at a constant level. Under such assumptions, the essential remodeling and homeostasis equations under consideration are as follows:

(3) $$\text{TENSIONAL HOMEOSTASIS: }\frac{dc}{dt}=\alpha \left(\sigma_{\theta \theta }^{\text{cell}}-\sigma_{\theta \theta }^{\text{target}}\right)$$

(4) $$\text{FILTRATION HOMEOSTASIS: }P^{\text{capillary}}=P^{\text{urine}}+\frac{\mu^{\text{plasma}}v_{r}^{\text{target}}h^{\text{GBM}}}{K^{GBM}}+P^{\text{oncotic}}$$

where c is the collagen volume fraction, t is time, α is a constant describing the rate of remodeling, σ^cell^ is the stress in the podocyte (outermost) layer of the model, σ^target^ is the podocyte target stress, P^capillary^ is the pressure in the capillary, P^urine^ is the pressure in the urinary space, *μ*^*plasma*^ is the plasma viscosity,$v_{r}^{\text{target}}$ is the target filtration velocity,*h*^*GBM*^ is the GBM thickness, K^GBM^ is the GBM permeability, and P^oncotic^ is the capillary oncotic pressure, that is, the osmotic pressure difference between the plasma and the urine, largely due to albumin. Target stress is defined implicitly as that at which tensional homeostasis is achieved, i.e. there are no differences in mechanical stress σ^cell^ − σ^target^ to drive remodeling, and dc/dt = 0; it is not necessarily fixed but adjustable in response to changes in the mechanobiological environment (Gyoneva et al. 2016; for further discussion, see Taber 2008). In our formulation of the remodeling equations, any factor that changes the remodeling process (e.g., partial production of the major chain in thin basement membrane disease) would be manifest as a change in the target stress; a more detailed model of the remodeling process could be used to explore such effects.

For all calculations in this manuscript, the target filtration velocity was set to 3.5 × 10^−6^ m/s, the plasma viscosity was set to 1 × 10^−3^ Pa•s, and the oncotic pressure was set to 25 mmHg (Barocas et al. 2012). P^urine^ was set to 15 mmHg. The cell target stress was set to arbitrarily to 45 Pa, which yielded reasonable results in the model and served to illustrate the principles in question; the effect of changing the cell target stress will be considered in the Discussion section.

One could, in principle, solve the two equations simultaneously to compute the predicted dynamic behavior of the coupled pressure-stress system, but for computational efficiency, we chose instead to focus on the steady-state condition for each equation separately for different amounts of deposited material. That is, since σ^cell^ is a function of P^capillary^, we examined what values of P^capillary^ would be consistent with a given target stress and amount of deposited collagen. Concurrently, for a given amount of deposited material, we determined what value for P^capillary^ would be necessary to achieve the desired filtration rate.

The overall methodology for drawing the tensional and filtration homeostasis curves was thus as follows. First, a collagen volume fraction was specified, and random networks with the appropriate volume fraction were constructed for all Gauss points in all elements of the simulated GBM. Next, the capillary pressure was increased progressively in the simulation, and at each pressure value, the cell stress and the matrix permeability were calculated as described above. The matrix permeability was then used to calculate the filtration rate at the applied pressure. This process continued until the pressure had reached a level that produced cell stress and filtration rate in excess of the target values, and the homeostatic pressure for each equation was interpolated from the results. Once the filtration-homeostasis and tensionalhomeostasis pressures for a given collagen volume fraction had been determined, a new collagen volume fraction was specified, and the process was repeated.

**Figure 2.**
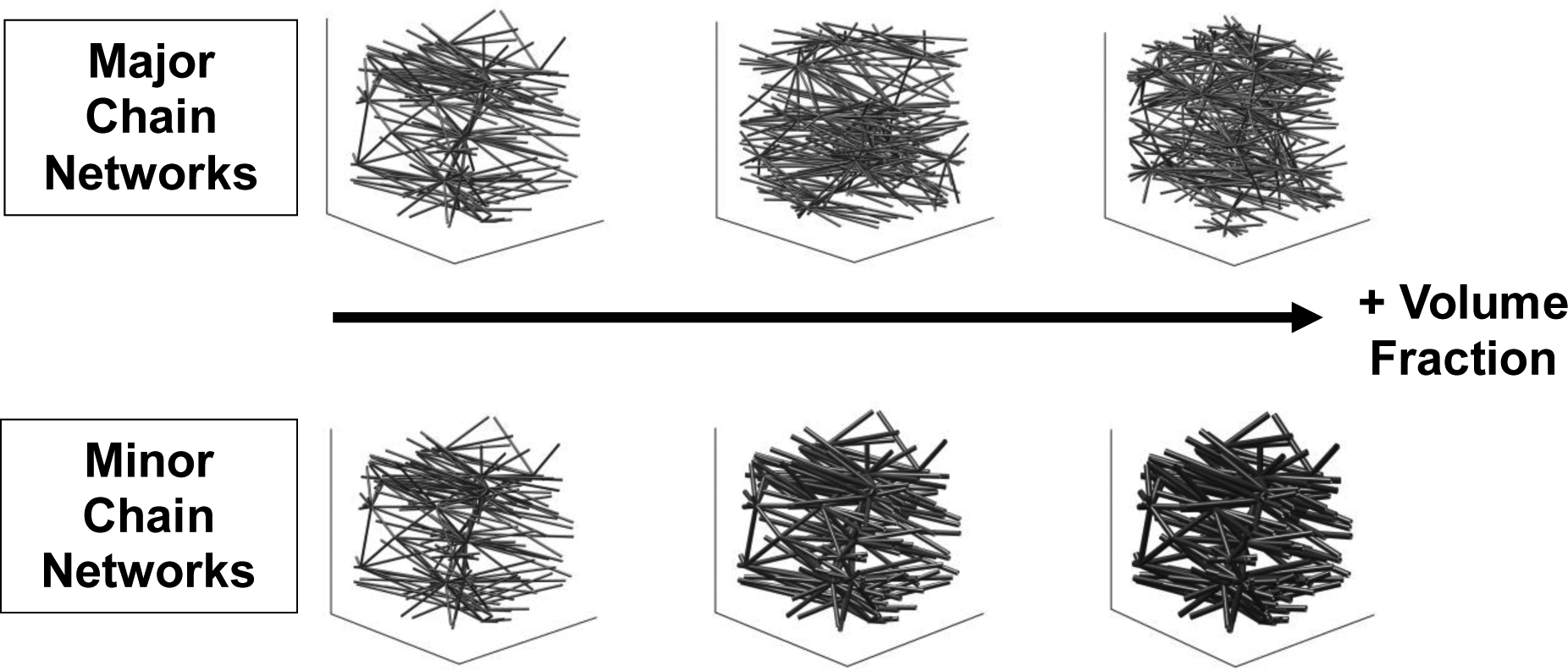
Model of Major-Chain vs. Minor-Chain Deposition. Since the major chain (upper row) does not supercoil significantly, volume was added to a major-chain network by increasing the number of fibers. For minor-chain networks (lower row), however, volume was added by increasing fiber radius, representing the new collagen supercoiling with fibers in the existing matrix.

Both minor-chain-deposition (healthy) and major-chain-deposition (Alport) models were used for the middle layer of the capillary wall in all studies. As noted above, a key feature of the minor chain is its ability to form stable supercoils that are not formed by the major chain. To represent this distinction, we modeled deposition of the major chains by adding new fibers to the model network, and that of the minor chains by increasing the diameter of existing fibers (Figure 2). In both cases, the resulting new networks were then used both for mechanical calculations and permeability calculations.

## Results

**Figure 3.**
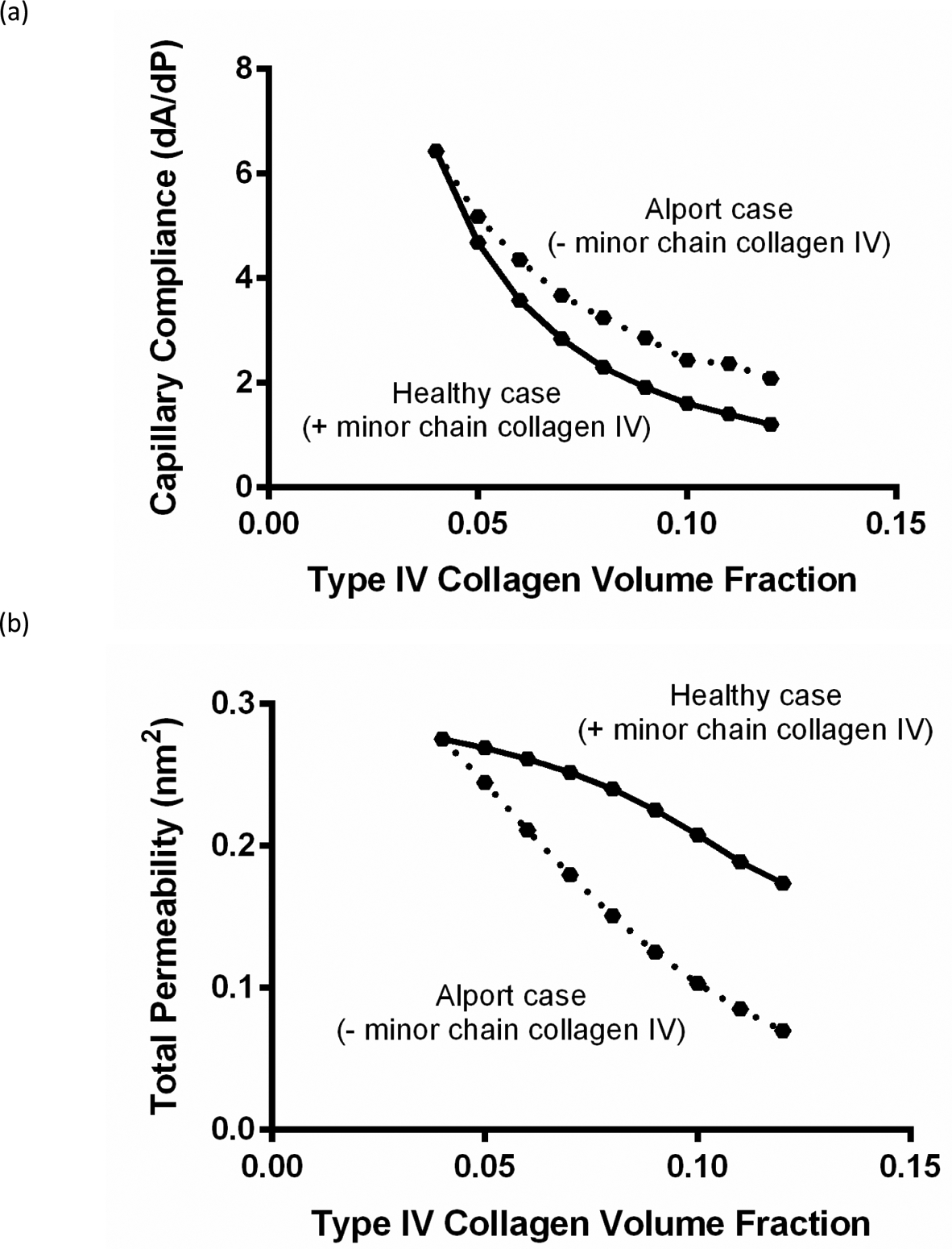
GBM Material Property Changes with Collagen Volume Fraction. (a) Compliance decreases (i.e., stiffness increases) with increased collagen level in both cases, with the healthy case showing a slightly stronger effect. (b) Permeability decreases with increased collagen level in both cases, but the Alport case shows a much larger decrease.

Figure 3 shows the changes in glomerular capillary compliance (Figure 3a) and permeability (Figure 3b) with increasing collagen content; an unremodeled 4% collagen fraction was taken as a starting point and thus gave the same values for both the healthy and the Alport cases. As expected, the deposition of more collagen makes the GBM stiffer and less permeable, a result that is both intuitive and consistent with our previous study (Gyoneva et al. 2015). There are, however, important differences between the two cases. First, as seen in Figure 3a, the healthy tissue stiffens (i.e., loses compliance) more quickly with collagen volume fraction; the more rapid stiffening means that the stress shielding by the collagen network - that is, reduction in the stress and strain experienced by the podocytes because the stiff collagen network resists deformation - is more effective in the healthy case. Second, as seen in Figure 3b, although both cases produce a loss in permeability with increased collagen content, the healthy remodeling process produces a much slower drop than in the Alport case. The differences between the healthy and Alport cases emphasize the importance of the minor chain in allowing the GBM to maintain its stiffness-permeability balance.

**Figure 4.**
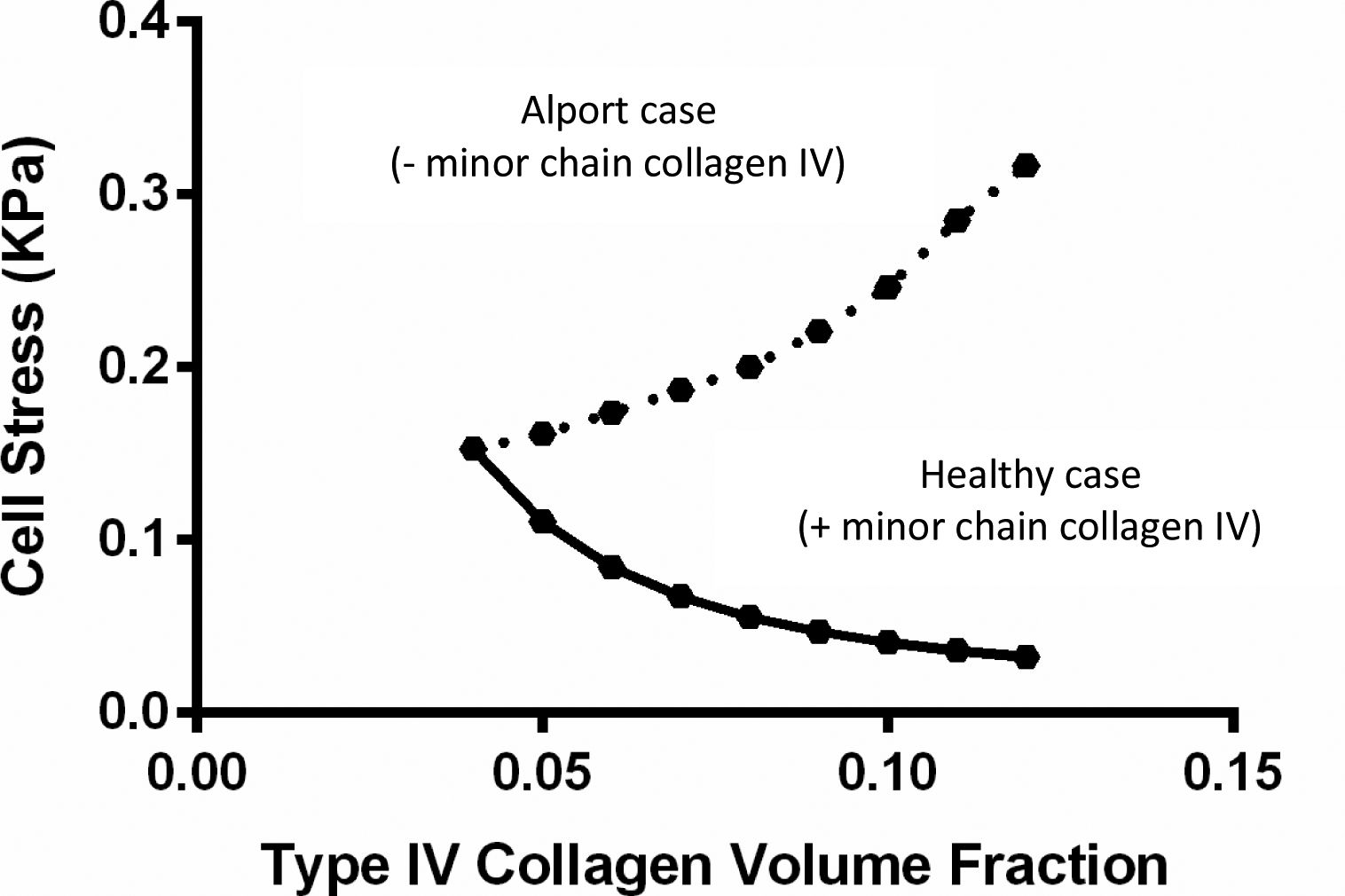
Podocyte Tension Changes with Collagen Volume Fraction. In the Alport case, the addition of more collagen drives *up* the cell stress because the increased pressure required to overcome the decreased permeability has more impact on cell stress than the increased shielding due to a stiffer matrix. For the healthy case, the shielding effect is stronger than the permeability/pressure effect, so the net effect is to drive *down* the cell stress at higher collagen levels.

Since the GBM stiffness increases monotonically with collagen density, one might suspect that depositing more collagen would provide greater shielding for the cells and thus reduce the cell stress. In the Alport case, however, the need for filtration homeostasis causes a counterintuitive increase in cell stress with increased collagen density, as shown in Figure 4. The driving cascade is as follows:

1. Increased collagen density leads to a marked decrease in GBM permeability, as shown in Figure 3b.
2. Because of the decreased permeability, a higher capillary pressure is required to maintain filtration homeostasis.
3. The increase in capillary pressure has a stronger effect than the increase in GBM stiffness, with the net result being an increase in podocyte stress. Thus, although the stiffening of the GBM causes the cells to carry a smaller fraction of the tissue load, the total tissue load increases because of the need for a higher filtration pressure.

**Figure 5.**
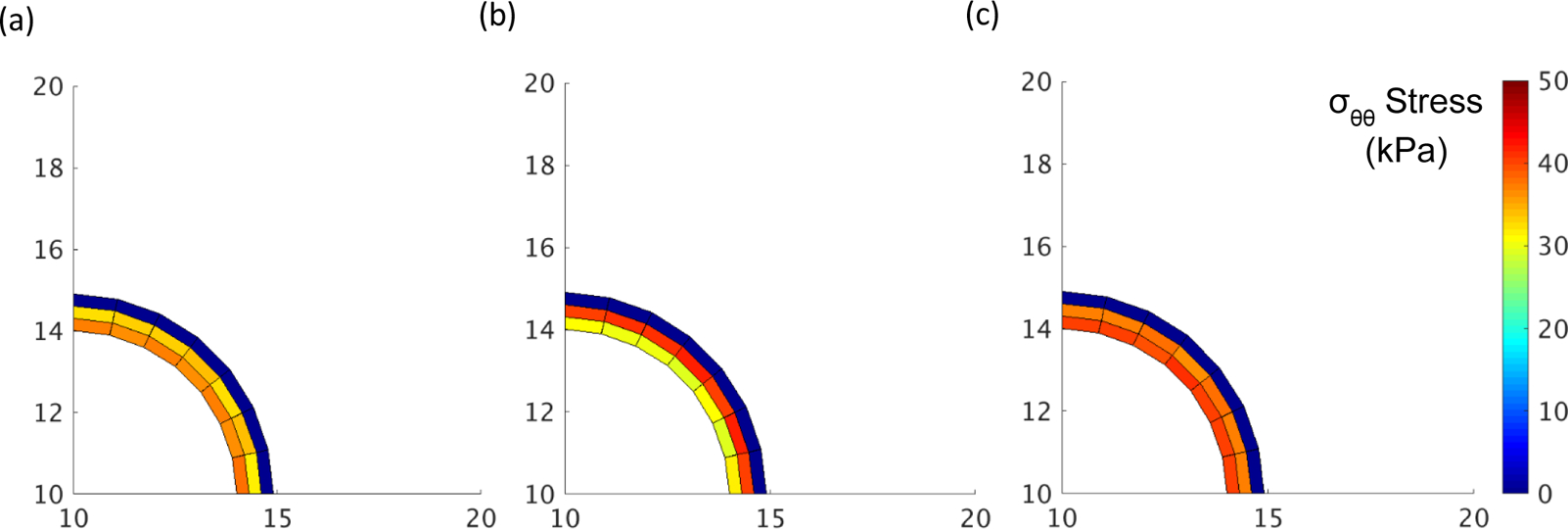
Circumferential stress (σ_θθ_) in the different layers of the simulated glomerular capillary wall. (a) Baseline (φ_0_ = 0.04). (b) Remodeled healthy (φ = 0.06). (c) Remodeled Alport (φ = 0.06). Colorbar is the same for all three cases. The remodeled healthy case (b) shows a slightly higher collagen stress due to the increased pressure necessary for filtration but a lower cell stress as the stiffer collagen layers protect the cells. In the Alport case (c), in contrast, the large drop in permeability (Figure 4b) leads to increased stress in both the collagen and the cell layers.

In the healthy case, the same effects arise, but the balance between them is shifted because of the different material property responses shown in Figure 3. Figure 5 shows circumferential stress distributions for an unremodeled GBM and for both the healthy and Alport cases with a 50% increase in the collagen density. in all cases, the cell stress is significantly lower than the GBM stress because of stress shielding by the stiffer GBM. In the healthy case (Figure 5b), the stress in the GBM increases and provides additional shielding for the podocytes in spite of an increase in capillary pressure. In the Alport case (Figure 5c), in contrast, a large increase in capillary pressure, arising due to the low permeability of the dense fiber matrix, leads to a net increase in podocyte pressure in spite of increased GBM stiffness.

**Figure 6.**
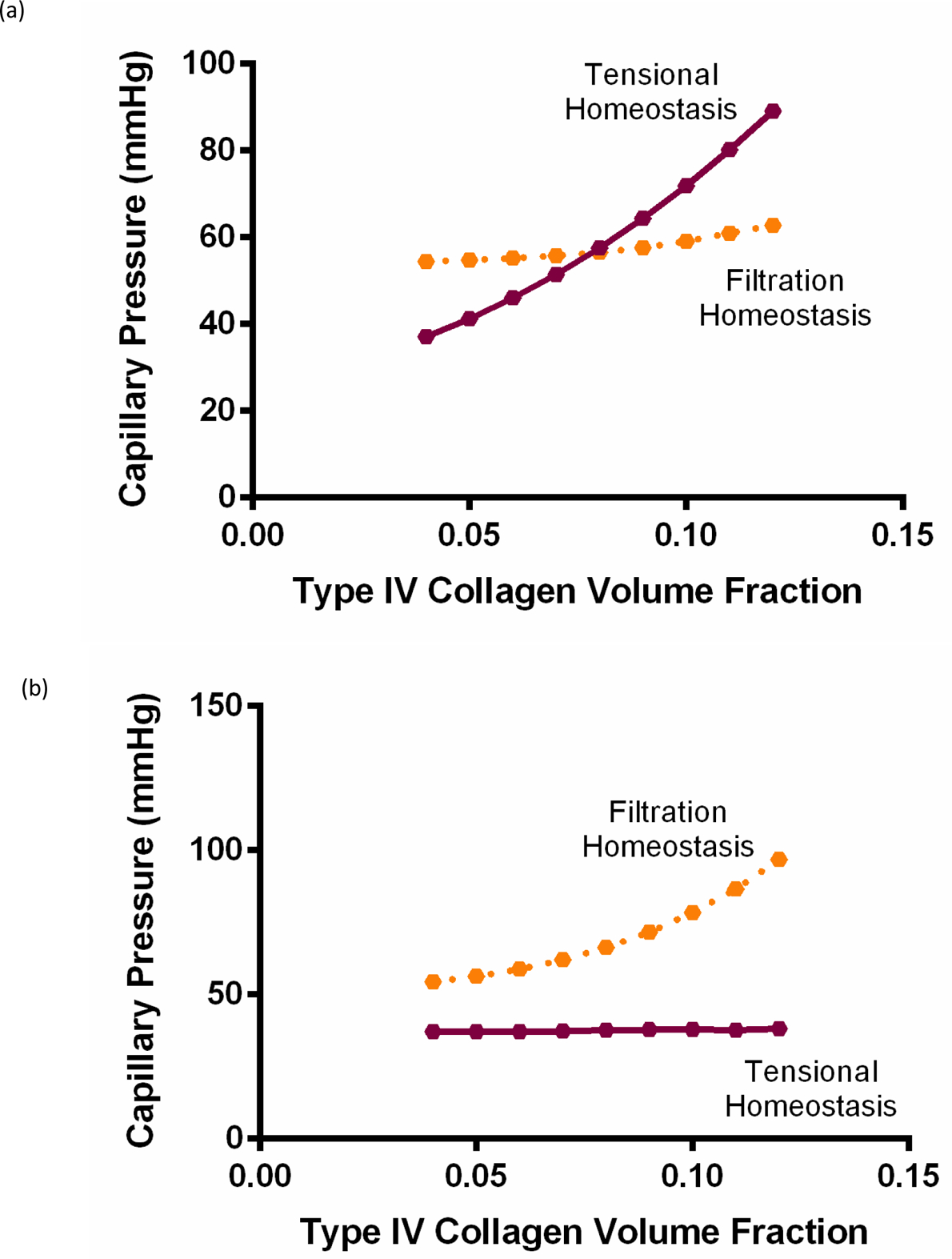
Capillary Pressure - Collagen Volume Fraction Operating Curves. (a) Healthy Case. There are two lines, one (filtration homeostasis) describing the pressure necessary to maintain the target filtration rate for a given collagen level, and the other (tensional homeostasis) describing the necessary collagen level to produce a tissue stiff enough to achieve the target cell stress at a given capillary pressure. Equilibrium is achieved where the two lines cross. (b) Alport Case. The lines have the same meaning, but now the filtration line is both higher and steeper, leading to no stable equilibrium condition.

The natural question, then, is whether the two homeostasis equations can be satisfied simultaneously, or what range of target stresses is compatible with filtration requirements. To explore this question, it is convenient to plot two curves of capillary pressure vs. GBM collagen density. The first, the *filtration homeostasis line*, indicates the pressure required to maintain filtration at a given collagen density; as the collagen density increases, the required pressure also increases because of decreased permeability. The second curve is the *tensional homeostasis line*, indicating the capillary pressure that can be tolerated by the cells at a given collagen density; again, as the collagen density increases, the allowable pressure increases because the stiffer matrix provides more effective stress shielding. These two lines can be generated from our results as shown in Figure 6, first for the healthy case (Figure 6a) and then for the Alport case (Figure 6b). In both cases, the filtration and tensional homeostasis lines both increase monotonically, but their slopes are quite different. For the healthy case, the tensional homeostasis line is steeper than the filtration homeostasis line, indicating that an increase in collagen volume fraction has a stronger effect on the GBM stiffness (and thus the podocytes’ ability to shield themselves from high stresses) than it does on the flow resistance. Where the two lines cross, both tensional and filtration homeostasis can be maintained, and the system can achieve equilibrium. In the Alport case of Figure 6b, however, the slope of the tensional homeostasis line is much lower than that of the filtration homeostasis line, indicating that adding new collagen leads to a greater increase in capillary pressure than in GBM stiffness. The two lines do not cross, and an equilibrium state Is unattainable.

In fact, the condition of Figure 6b would be fundamentally different from that of Figure 6a even if the lines did cross, because changing the slopes of the two lines changes their intersection point from a stable to an unstable equilibrium. This can be seen readily by assuming a locally linear response in which (1) the pressure *P* equilibrates to maintain filtration, so P = P_F_(c), where P_F_ is the filtration curve from Figure 6, and (2) the collagen volume fraction *c* evolves linearly based on deviation from its tensional target, comparable to equation (3) but expressed in terms of target collagen volume fraction instead of stress:

(5) $$\frac{dc}{dt}=\beta \left(c_{T}\left(P_{F}(c)\right)-c\right)$$

where c_T_ is the collagen volume fraction required to achieve tensional homeostasis for a given P, as described by the tensional line in Figure 6. The stability of this model is analyzed by differentiating the equation with respect to c:

(6) $$\frac{d}{dc}\left(\frac{dc}{dt}\right)=\beta \left({\left[\frac{dc}{dP}\right]}_{T}{\left[\frac{dP}{dc}\right]}_{F}-1\right)$$

where [•]_T_ and [•]_F_ refer to derivatives taken along the tensional and filtration homeostasis lines, respectively. Since [dc/dP]_T_ is the inverse of the slope of the tensional line in Figure 6, and [dP/dc]_F_ is the slope of the filtration line in Figure 6, $\frac{d}{dc}\left(\frac{dc}{dt}\right)$ depends on the relative slopes of the two lines. If the tensional line has a greater slope than the filtration line (as in the healthy case of Figure 6a), $\frac{d}{dc}\left(\frac{dc}{dt}\right)$ is negative, indicating stability of the system. If, however, the filtration line has greater slope (as in the Alport case of Figure 6b), $\frac{d}{dc}\left(\frac{dc}{dt}\right)$ is positive, indicating instability.

## Discussion

Glomerular capillary remodeling is constrained by the need to maintain permeability to allow the kidney to filter blood. The minor collagen chain network, which is prominent in the kidney glomerulus and more so than nearly anywhere else in the body (Miner and Sanes 1994; Peissel et al. 1995), appears uniquely suited to meet this twofold challenge, and the present work has demonstrated how the interaction between the two homeostatic driving forces can yield either a stable or an unstable system depending on the mechanism by which remodeling occurs. The model presented herein simplifies many aspects of both filtration and remodeling but draws attention to the importance of considering multiple factors driving the remodeling process and to the need for better understanding of how different driving forces interact. Particularly when one considers vascular and other basement membranes, there are often multiple, potentially competing driving forces at work. Other examples include extreme selectivity maintained by the blood-brain barrier while allowing a high degree of oxygen transport and responsiveness to regional metabolic demands (Iadecola 2017); the need for lung capillaries to allow rapid oxygen transport but also withstand high capillary pressures, especially during exercise (West 2000); and the lens capsule basement membrane, which must allow mechanical control of lens shape by the ciliary muscle and also allow nutrient and waste transport for lens cells (Mathias et al. 1997).

For the model used and the parameters chosen, we found that the Alport case is not able to achieve equilibrium. Although we did not run dynamic simulations in this study, it is clear from the results that the model would predict the collagen density and the cell stress to increase without bound since the cell could never reduce its stress to the target level. This result is consistent with the focal characteristic GBM thickening in Alport syndrome (Rumpelt et al. 1974). Alport syndrome is characterized by irregularity of GBM thickness and progression from normal GFR to eventual delamination failure of the GBM, also dynamic effects not addressed by the current model but potentially explored in future work.

In considering the plots of Figure 6, one can see that various phenomena would shift one line or another. For example, a decrease in filtration demand would shift the filtration homeostasis line downward (lower pressure needed). This particular effect is notable because Alport syndrome is generally asymptomatic prior to adolescence, and it may be that lowering filtration demand during youth would shift the filtration homeostasis line in Figure 6b to a low enough position that equilibrium is possible. Even in that case, though, the equilibrium would be unstable, so the disease would be expected to progress, although perhaps less quickly. Similarly, our choice of the target stress was arbitrary. Increasing the target stress to 100 Pa would shift the tensional homeostasis curve up considerably, resulting in a target type IV collagen volume fraction of only about 4% in the healthy case (vs. about 8% in Figure 6a), and bring the two lines much closer together in the Alport case. Combining an increase in target stress with a reduction in filtration demand would cause the two lines to cross in the Alport case, but the resulting equilibrium would still be unstable.

Alport syndrome is progressive and irreversible, with patients eventually requiring dialysis or transplantation. Current therapeutic strategies slow but do not prevent progression to kidney failure (Gross et al. 2020). Among established treatments, inhibition of the renin-angiotensin system may exert multiple effects, decreasing glomerular capillary pressure directly and thereby cell stress, increasing glomerular capillary surface area and attainment of filtration homeostasis at lower capillary pressure, and facilitating podocyte adaptation to the mechanical environment. Insights from this work suggest other ways to think about treatment. First, new approaches could be focused on mechanoregulatory pathways. For example, if one could deliver a drug that alters the podocytes’ mechanobiological machinery, which would lead to a change in the target stress of equation (3), one might be able to shift the tensional homeostasis line in Figure 6 and thus allow equilibrium to be achieved or maintained (Endlich et al. 2017), particularly if the slope of the curve could be modified to bring the system into a stable condition. While unexplored in Alport syndrome, drugs that act through mechanoregulatory pathways have been under early investigation in general (Kothari et al. 2019; Surcel and Robinson 2019) and with respect to podocyte disorders (reviewed in (Müller-Deile and Schiffer 2016; Sever and Schiffer 2018)). Second, absent options for gene replacement of the minor chain, it may be possible to target glomerular major chain remodeling favorably, effectively modifying the rate constant *α* in equation (3), so as to slow progression of Alport syndrome. Such options may be afforded, for example, by lysis oxidase inhibitors, which demonstrate partial benefits in a mouse model of Alport syndrome (Cosgrove et al. 2018) and whose key effects include those on type IV collagen (Añazco et al. 2016). Third, it might be possible to shift the filtration homeostasis line, for example through modification of intrinsic GBM properties or change in glomerular capillary surface area, thereby decreasing the capillary pressure by equation (4) and the stimulus for collagen production. Such may emerge as one mechanism of action for bardoxolone, an agent that is currently under clinical investigation for Alport syndrome (Baigent and Lennon 2018; Toto 2018). Finally, inasmuch as filtration homeostasis is itself determined by needs for solute and water excretion, dietary approaches that draw on a historic body of research in kidney diseases (Kalantar-Zadeh and Fouque 2017; Yu et al. 2019; Goraya and Wesson 2020), may have roles in Alport syndrome. Collectively, potential therapeutic approaches such as these and others may offer significant benefits by slowing the dynamics of disease progression in Alport syndrome, even if instability of the system remains.

In conclusion, we recognize that our model is a simplification of the highly complex processes involved in GBM remodeling, but it provides a new way of thinking about Alport syndrome - as the emergence of an unstable remodeling process - which can inform new thinking about treatment for Alport syndrome and glomerular disease more broadly.

## Figures and Tables

**Table 1.** Parameter values used for the fiber constitutive equations in the microstructural network. For the minor-chain diameter, the value given is the initial value prior to any remodeling.

Parameter	Value
**r**_**major chain**_	7 nm
**r**_**minor chain**_	3.5 nm
**r**_**podocyte**_	0.7 nm
**A**	πr^2^ nm^2^
**E**_**collagen**_	5 × 10^9^ Pa
**E**_**podocyte**_	2.5 × 10^9^ Pa
**B**	0.25
**λ**_**limit**_	1.3
